# Unique Proteomic Signatures Distinguish Macrophages and Dendritic Cells

**DOI:** 10.1371/journal.pone.0033297

**Published:** 2012-03-12

**Authors:** Lev Becker, Ning-Chun Liu, Michelle M. Averill, Wei Yuan, Nathalie Pamir, Yufeng Peng, Angela D. Irwin, Xiaoyun Fu, Karin E. Bornfeldt, Jay W. Heinecke

**Affiliations:** 1 Department of Medicine, University of Washington, Seattle, Washington, United States of America; 2 Department of Pathology, University of Washington, Seattle, Washington, United States of America; 3 Department of Rheumatology, University of Washington, Seattle, Washington, United States of America; 4 Diabetes and Obesity Center of Excellence, University of Washington, Seattle, Washington, United States of America; Ulm University, Germany

## Abstract

Monocytes differentiate into heterogeneous populations of tissue macrophages and dendritic cells (DCs) that regulate inflammation and immunity. Identifying specific populations of myeloid cells *in vivo* is problematic, however, because only a limited number of proteins have been used to assign cellular phenotype. Using mass spectrometry and bone marrow-derived cells, we provided a global view of the proteomes of M-CSF-derived macrophages, classically and alternatively activated macrophages, and GM-CSF-derived DCs. Remarkably, the expression levels of half the plasma membrane proteins differed significantly in the various populations of cells derived *in vitro*. Moreover, the membrane proteomes of macrophages and DCs were more distinct than those of classically and alternatively activated macrophages. Hierarchical cluster and dual statistical analyses demonstrated that each cell type exhibited a robust proteomic signature that was unique. To interrogate the phenotype of myeloid cells *in vivo*, we subjected elicited peritoneal macrophages harvested from wild-type and GM-CSF-deficient mice to mass spectrometric and functional analysis. Unexpectedly, we found that peritoneal macrophages exhibited many features of the DCs generated *in vitro*. These findings demonstrate that global analysis of the membrane proteome can help define immune cell phenotypes in vivo.

## Introduction

Monocytes emigrate from blood vessels into tissue, where they differentiate into a variety of specialized macrophage populations central to tissue homeostasis, immunity, and inflammation [1]. Thus, macrophages exhibit marked phenotypic heterogeneity *in vitro* and *in vivo* [2], [3]. Based on patterns of gene expression, protein secretion, and function, they have been classified as classically activated (M1) or alternatively activated (M2) cells. The M1 phenotype is promoted by Th1 mediators such as LPS and IFN-γ and is characterized by the production of pro-inflammatory cytokines and nitric oxide (NO) and by potent anti-microbial activity [4]. In contrast, the M2 phenotype is induced by Th2 mediators, typically IL-4 or IL-13 [5], and it triggers expression of arginase, proteinases, and immunosuppressive factors. Hence, alternatively activated macrophages participate in tissue remodeling after injury and help resolve inflammation [5].

Monocytes also can differentiate into myeloid dendritic cells (DCs), which play specialized roles in host defense and antigen presentation [6]–[8]. Populations of myeloid DCs that express high levels of TNFα and iNOS (Tip DCs) have been identified in inflamed tissues [8], and they mediate innate defense against bacterial pathogens. Moreover, recent studies demonstrate that monocyte-derived cells localize to T cell areas of lymph nodes to cross-present antigen, indicating that monocytes can develop into cells that exhibit the critical immune features of DCs *in vivo* [6].

It is currently difficult to distinguish DCs from macrophages because there is little agreement about the utility of specific markers for identifying distinct cell types in tissues [9]. Commonly, only a limited number of proteins have been used to assign cellular phenotype, and the specificity of such proteins for any given subpopulation has not been rigorously demonstrated [9]. Mass spectrometry has been widely used to investigate the proteomes of macrophages and dendritic cells [10]–[12], but much less is known about the plasma membrane proteomes of these cells. Moreover, no studies have directly compared the proteomes of macrophages and DCs.

In the current studies, we used mass spectrometry to probe the plasma membrane proteomes of macrophages, polarized macrophages, and DCs. We used bone marrow-derived cells because differentiation *ex vivo* permits the generation of relatively homogeneous, well-characterized cell populations that have been studied by many investigators. To investigate the physiological significance of the *in vitro* cells, we also determined the membrane proteomes of elicited peritoneal macrophages harvested from wild-type and GM-CSF-deficient mice. We found that macrophages and DCs have different membrane proteomes and that their protein expression patterns distinguish them *in vivo* as well as *in vitro*.

## Results

### Tandem mass spectrometry (MS/MS) identifies proteins that are greatly enriched in the plasma membrane of myeloid cells

We focused our mass spectrometric studies on the plasma membrane proteomes of macrophages and DCs for two reasons. First, membrane proteins serve critical roles in a variety of functions characteristic of this family of leukocytes, such as phagocytosis, cell migration, and antigen presentation. Second, plasma membrane proteins are well-positioned for flow cytometric analysis, which is widely used to isolate and interrogate the phenotypes of specific populations of immune cells *in vivo*.

**Figure 1:**
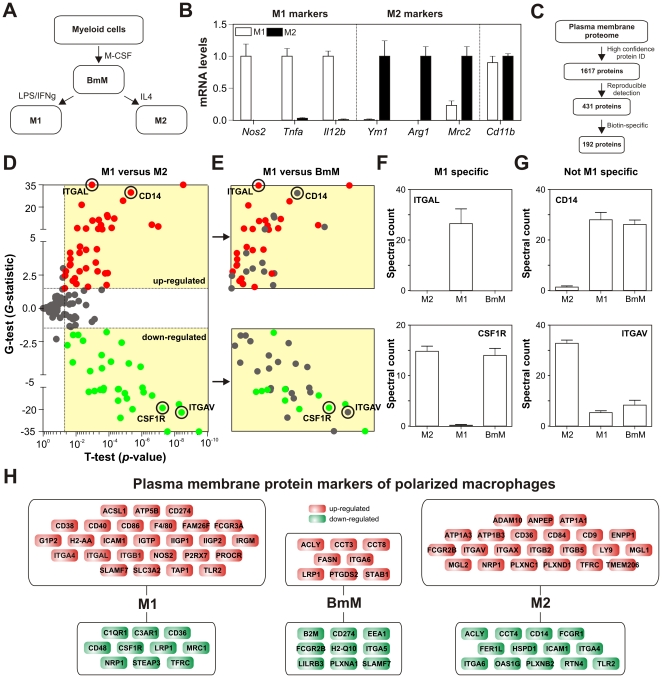
The plasma membrane proteome of macrophages. ***Panel A:*** Bone marrow-derived macrophages (BmM) were derived from bone marrow precursor cells of C57BL/6 mice cultured with M-CSF. Classically activated macrophages (M1) and alternatively activated macrophages (M2) were derived from BmMs by treatment with IFN-γ and LPS or with IL-4. ***Panel B:*** qRT-PCR of markers used to detect M1 and M2 macrophages. Results (means and SEMs, N = 6) were standardized to 18S, expressed relative to the cell type with the highest expression of each gene, and are representative of 3 independent analyses. ***Panel C:*** LC-ESI-MS/MS analysis of plasma membrane proteins isolated from differentially activated macrophages. Proteins were quantified by spectral counting (total number of peptides identified for a given protein) and subjected to sequential criteria to identify 192 plasma membrane proteins that were reproducibly detected with high confidence. ***Panel D:*** Quantification of the membrane proteomes of M1 and M2 macrophages. Differentially expressed proteins (red, upregulated; green, downregulated; gray, not significantly different) were identified based on *t-*test and *G-*test statistics. Significance cutoffs (dashed lines; *p*<0.05 and *G-*statistic >1.5 or <−1.5) were determined based on permutation analysis (estimated FDR<5%). ***Panel E:*** Quantification of the membrane proteomes of M1 macrophages and BmMs. Proteins differentially expressed by M1 cells relative to both BmMs and M2 cells are indicated with colored dots (red, upregulated; green, downregulated). Proteins differentially expressed by M1 and M2 cells (*Panel D*) but not differentially expressed by M1 and BmMs are indicated by gray dots. ***Panel F:*** Examples of proteins that distinguish M1 cells from both BmM and M2 cells (CSF1R, ITGAL). Results (N = 6 per group) are means and SDs. ***Panel G:*** Examples of proteins that fail to distinguish M1 cells from both BmM and M2 cells (CD14, ITGAV). ***Panel H:*** Plasma membrane proteins differentially expressed by M1 cells (36 proteins), M2 cells (35 proteins), and BmMs (17 proteins).

To generate large numbers of macrophages (**Fig. 1A**), we incubated bone marrow cells from C57BL/6J mice with colony stimulating factor-1 (M-CSF; gene name *Csf1*). We term the resulting adherent cells, which were harvested after 6 days in culture, bone marrow-derived macrophages (BmMs). BmMs were polarized into M1 or M2 macrophages by exposure for 24 h to IFN-γ/LPS or IL-4, respectively. Differentiation to M1 or M2 macrophages was confirmed by using qRT-PCR to interrogate the cells for expression of accepted M1-specific (*Nos2*, *Tnfa*, *Il12b*) and M2-specific (*Ym1*, *Arg1*, *Mrc2*) genes (**Fig. 1B**) [2], [13]. The model system we used to generate macrophages and DCs (below) was reproducible and has been used by many investigators.

Membrane-associated proteins were biotinylated, affinity-isolated, and analyzed with LC-ESI-MS/MS. The resulting tandem MS data were processed using three independent criteria (**Fig. 1C**). To ensure high-confidence protein identification, we used two Bayesian algorithms (PeptideProphet [14] and ProteinProphet [15]). To select for reproducible protein detection, we required that each protein be detected in at least 5 of 6 biological replicates in at least one cell type. To control for nonspecific interactions with the biotin-affinity column, we excluded proteins detected in samples prepared from unlabeled cells.

This approach identified 192 cell membrane-associated proteins with high confidence (**Table S1**). Gene ontology analysis revealed that 77% (148 of 192) of the proteins were known membrane-associated proteins (*p* = 10^−15^) and that more than half of those (79 of 148) had been previously localized to the plasma membrane (*p* = 10^−19^), indicating that our biochemical approach yielded a subproteome that was greatly enriched in plasma membrane proteins. The top three molecular functions were triphosphatase activity (*p* = 10^−10^), GTPase activity (*p* = 10^−6^), and ATP-dependent transport (*p* = 10^−5^), while the top three biological processes were antigen processing (*p* = 10^−10^), endocytosis (*p* = 10^−7^), and cell adhesion (*p* = 10^−6^), which are strongly linked to membrane activities critical to immune cells. In contrast, gene ontology analysis of proteins excluded by our analytical criteria revealed that these contaminants corresponded to abundant cytoskeletal (*p* = 10^−11^) and cytosolic (*p* = 10^−6^) proteins involved in housekeeping functions such as actin-cytoskeleton organization (*p* = 10^−10^), translation (*p* = 10^−11^), and glycolysis (*p* = 10^−9^).

### Dual statistical analyses identified a wide range of plasma membrane proteins that are differentially expressed by polarized macrophages

The plasma membrane proteome of BmMs contained numerous proteins (e.g., F4/80, CD14, CD11b, and CD11c) that are commonly used as macrophage-specific markers, both *in vitro* and *in vivo*. However, the specificity of many of these markers has been questioned [9]. To identify proteins that accurately distinguish the various macrophage subtypes generated *in vitro*, we quantified proteins by spectral counting (a measure of relative protein concentration [16]) and analyzed the data using both the *t-*test and *G-*test [17], [18].

Our approach to defining protein expression patterns that are specific to a particular cell phenotype is illustrated for M1 macrophages (**Fig. 1D–F**). We first compared protein expression levels in M1 and M2 macrophages (**Fig. 1D**). This analysis identified 41 proteins expressed at higher levels and 33 proteins expressed at lower levels in M1 cells than in M2 cells (*t-*test, *p*<0.05; *G-*test, *G-*statistic >1.5 or <−1.5; FDR = 2.5%). We next required that, for a given protein to be a marker of the M1 phenotype, its level also had to significantly differ between M1 macrophages and BmMs. This additional constraint eliminated more than half of the 74 proteins that tentatively distinguished M1 from M2 macrophages (**Fig. 1E**). For example, ITGAL and CSF1R were M1-specific (**Fig. 1F**) while CD14 and ITGAV were not (**Fig. 1G**). Using this approach for all possible pairwise comparisons of cells, we identified 86 proteins that distinguished the various types of macrophages from each other (**Fig. 1H**; 17 protein for BmMs; 36 proteins for M1 cells; 35 proteins for M2 cells; **Table S2**).

### Hierarchical cluster and dual statistical analyses demonstrate that macrophages and DCs have different membrane proteomes

**Figure 2:**
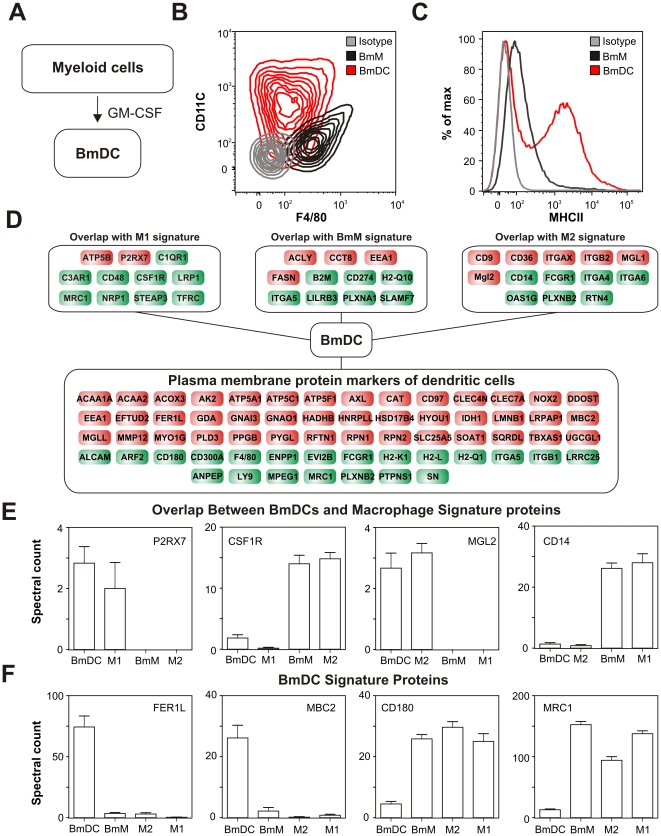
The plasma membrane proteome of bone marrow-derived dendritic cells (DCs). ***Panel A:*** Bone marrow-derived dendritic cells (BmDCs) were obtained by culturing bone marrow cells with GM-CSF. ***Panel B:*** Flow cytometric analysis of CD11c and F4/80 expression in BmDCs and BmMs. Results are presented as a contour plot with 10% probability increments. ***Panel C:*** Cell-surface exp ression of MHC-II by BmDCs and BmMs as assessed by flow cytometry. ***Panel D:*** The plasma membrane proteome of DCs. *Upper Panel:* Proteins expressed at similar levels by DCs and either M1 cells, M2 cells, or BmMs. *Lower Panel:* Proteins differentially expressed by DCs relative to M1 cells, M2 cells, and BmMs (*G-*test>1.5 or <−1.5 and *t-*test: *p*<0.05). Red, upregulated; green, downregulated. ***Panel E:*** Examples of proteins expressed at similar levels by DCs and either M1 cells, or M2 cells. Results (N = 6 per group) are means and SDs. ***Panel F:*** Examples of plasma membrane proteins differentially expressed by DCs. Flow cytometry experiments are representative of 3 independent analyses.

Recent studies suggest that in addition to giving rise to macrophages, monocytes can also serve as precursor cells to specific populations of antigen presenting dendritic cells (DCs) [6]. To interrogate the plasma membrane proteome of DCs, we treated bone marrow precursor cells with GM-CSF (**Fig. 2A**) and performed tandem MS analysis on proteins isolated from their plasma membranes. Myeloid DCs (BmDCs) generated by culturing bone marrow cells with GM-CSF (gene name *Csf2*) are a model of immature DCs that share many functional characteristics with their *in vivo* counterparts [19]. Consistent with previous observations [19], [20], cell sorting analysis demonstrated that BmDCs had higher CD11c and MHC-II expression and lower F4/80 expression than BmMs (**Fig. 2B–C** **, Fig. S1**).

Analysis of the membrane proteome of BmDCs revealed that many of the proteins that appeared to be characteristic of BmMs or M1 or M2 macrophages (**Fig. 1D**) were also expressed at similar levels by BmDCs (**Fig. 2D** **, Table S3**). Examples of such proteins include P2RX7 and CSF1R for M1 cells and MGL2 and CD14 for M2 cells (**Fig. 2E**). Inclusion of the BmDC plasma membrane proteome, therefore, substantially refined the signatures identified for M1, BmM, and M2 macrophages (**Table S3**). In addition, BmDCs expressed a set of 63 proteins that distinguished them from the three macrophage subpopulations tested (**Fig. 2D**). FER1L, MBC2, CD180, and MRC1 are examples of such proteins (**Fig. 2F**).

These findings indicate that, while the plasma membrane proteomes of BmDCs and polarized macrophages overlap considerably, BmDCs express a large set of membrane proteins distinct from those of macrophages. To further support this conclusion, we compared the membrane proteomes of myeloid cells by hierarchical cluster analysis. This approach clearly differentiated the various cell types (**Fig. S2**). Importantly, all biological replicates *within* a given cell type were tightly clustered and completely segregated from biological replicates *across* cell types. Hierarchical cluster analysis further demonstrated that the membrane proteomes of macrophages and BmDCs were more distinct from one another than were those of M1 and M2 macrophages (**Fig. S2**).

### Immunocytochemical analysis validates candidate proteomic signatures of myeloid cells

**Figure 3:**
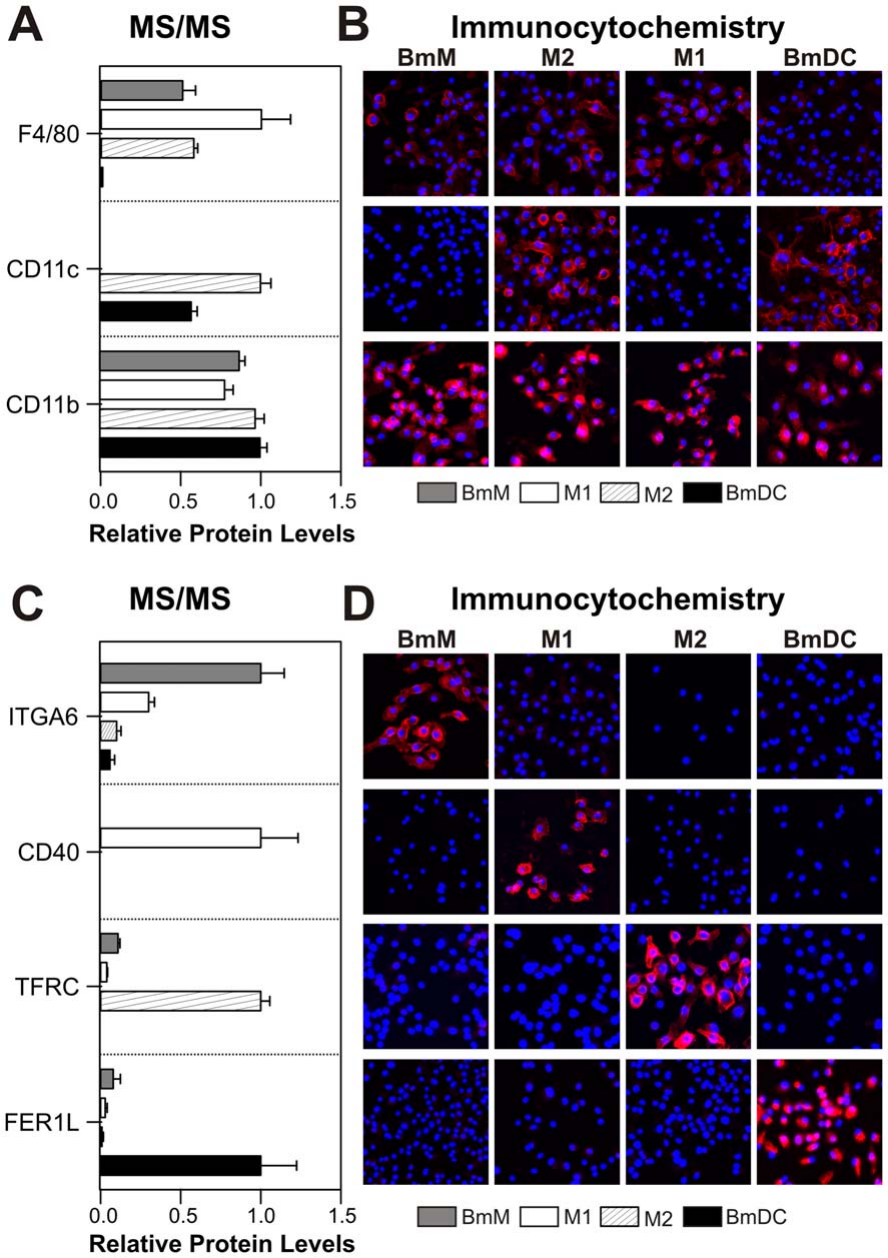
Immunocytochemical detection of plasma membrane protein markers. Expression levels of widely used plasma membrane protein markers (***Panels A–B***) and newly identified markers (***Panels C–D***) of M1 cells, M2 cells, BmMs, and BmDCs were assessed by mass spectrometry (***Panels A,C***) and immunocytochemistry (***Panels B,D***). For MS/MS, proteins were quantified by spectral counting and expressed relative to the cell type with the highest expression level for each protein. Results are means and SDs. Cells were stained with antibodies specific to each protein (red channel), counterstained with DAPI to visualize nuclei (blue-channel), and examined by confocal microscopy. Immunostaining and microscopy were performed on the same day with identical microscope settings. Results are representative of 3 independent analyses.

Our proteomic analysis identified a large number of plasma membrane markers that may identify myeloid cell phenotypes *in vitro* and *in vivo*. While some of these markers are already in use, most of the proteins that were enriched in the membranes of the various cell types have not been described as macrophage/DC markers. To validate our findings, we investigated the expression levels of a variety of candidate protein markers (both previously characterized and new) by mass spectrometry and immunocytochemistry (**Fig. 3** **, Fig. S3**).

In general, the two methods were in excellent agreement for the proteins we examined. As is well established, high levels of F4/80 protein were detectable on all macrophage subpopulations (BmMs, M1, and M2) but not on BmDCs (**Fig. 3A–B**). In addition, we found high levels of CD11c on both M2 macrophages and BmDCs (**Fig. 3A–B**). These data further support the notion that CD11c, often considered to be a DC marker, is not specific for those cells [9]. Moreover, strong CD11b expression was detected on the cell surface of all myeloid cells (**Fig. 3A–B**). With respect to novel markers, immunostaining confirmed the increased expression of the markers we identified by MS/MS (**Fig. 3C–D**, **Fig. S3**): the α_6_ integrin subunit (ITGA6) for BmMs; transferrin receptor (TFRC) for M2 cells; TNF receptor superfamily member 5 (CD40) for M1 cells; and extended synaptotagmin-1 (MBC2) for BmDCs.

### The plasma membrane proteomes of elicited peritoneal “macrophages” and BmDCs are remarkably similar

We next determined whether the proteomic signatures generated from myeloid cells derived *in vitro* predicted myeloid cell phenotypes *in vivo*. We therefore injected C57BL/6J-*Ldlr^tm1Her^* mice with thioglycolate, harvested elicited myeloid peritoneal cells (eMPCs) from the peritoneum 5 days later, isolated the aherent cells' plasma membrane proteins with affinity chromatography, and analyzed those proteins with LC-ESI-MS/MS.

**Figure 4:**
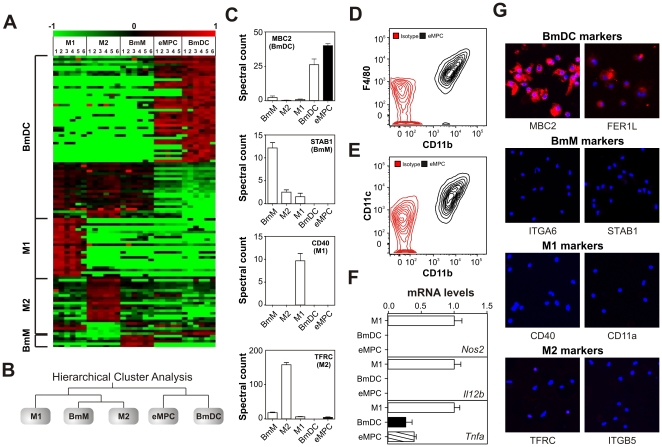
Mass spectrometric and immunohistochemical staining of thioglycolate-elicited peritoneal cells (eMPCs), polarized macrophages, and DCs. ***Panels A–B:*** Hierarchical cluster analysis of eMPCs. Cells were harvested from the peritoneal cavity of C57BL/6J-*Ldlr^tm1Her^* mice 5 days after intraperitoneal injection with thioglycolate. Isolated plasma membrane proteins detected by LC-MS/MS analysis of eMPCs were subjected to hierarchical cluster analysis, using the 107 proteins identified as differentially expressed by myeloid cells generated *in vitro* (**Fig. 2**). ***Panel B:*** Relationships among eMPCs, M1 cells, M2 cells, BmMs, and DCs, as determined by cluster analysis. ***Panel C:*** Protein expression in eMPCs, M1 cells, M2 cells, BmMs, and BmDCs. Protein levels were quantified by MS/MS and spectral counting. Data are presented as means and SDs. ***Panels D–E:*** Flow cytometric analysis of CD11b, CD11c, and F4/80 in eMPCs. Results are presented as contour plots with 10% probability increments. ***Panel F:*** qRT-PCR analysis of M1 marker genes (*Nos2*, *Il12b*, *Tnfa*) in M1 macrophages, BmDCs, and eMPCs. Results (means and SEMs; N = 6) were standardized to 18S levels and expressed relative to M1 macrophages. ***Panel G:*** Immunostaining of eMPCs. Cells were stained with antibodies (red channel) to plasma membrane proteins differentially expressed by BmDCs (MBC2, FER1L), BmMs (ITGA6, STAB1), M2 cells (TFRC, ITGB5), and M1 cells (CD11a, CD40). Nuclei were visualized by DAPI staining (blue channel). Immunostaining and microscopy were performed on the same day and with identical microscope settings to experiments presented in **Fig. 3D** and **Fig. S3**. Results obtained for flow cytometry, qRT-PCR, and immunocotyochemistry are representative of 3 independent analyses.

Our analyses focused on eMPCs, which are widely used in macrophage studies. Unexpectedly, we found that eMPCs expressed 64% (41 of 64) of the proteins that were enriched in BmDCs generated *in vitro* with GM-CSF. In contrast, eMPCs failed to express any of the 24 M1 cell markers or 22 M2 cell markers, and they expressed only 1 of the 5 BmM markers (**Fig. 4A**, **Table S4**). The expression levels (quantified by MS/MS) of representative membrane proteins are shown in **Figure 4C**. Hierarchical cluster analysis confirmed that eMPCs were similar to BmDCs and were distinct from the three macrophage types (**Fig. 4A–B**).

Our data indicate that eMPCs are more similar to BmDCs than any of the types of *in vitro* generated macrophage we investigated. Because MS/MS analysis quantifies the average protein expression level across the entire population of cells, we cannot exclude the possibility that the proteomic signature of eMPCs is comprised of a weight-averaged signal from a highly heterogeneous mixture of cells. To investigate this possibility, we examined CD11b, F4/80, and CD11c expression by flow cytometry, an approach previously used to assess cellular heterogeneity in this model system [21]. This approach demonstrated a single population of eMPCs (**Fig. 4D–E** **, Fig. S1**), suggesting that eMPCs harvested from C57BL/6J mice represent are reasonably homogeneous as assessed by these markers.

eMPCs are often regarded as “inflammatory macrophages” [22], [23]. We therefore compared the expression of pro-inflammatory cytokines by M1 macrophages, BmDCs, and eMPCs. We found that eMPCs and BmDCs expressed similar levels of *Nos2*, *Il12b*, and *Tnfa* mRNAs but that M1 macrophages expressed higher levels (**Fig. 4F**).

Immunocytochemical staining with a panel of 8 antibodies to markers of the different myeloid-derived cells demonstrated that eMPCs, like BmDCs, stained positive for FER1L and MBC2. In contrast, eMPCs and BmDCs did not react with antibodies for the macrophage markers CD11a, CD40, ITGA6, STAB1, TFRC or ITGB5 (**Fig. 4G**). Collectively, these findings indicate that eMPCs strongly resemble DCs generated *in vitro* with GM-CSF and are distinct from any macrophage population tested.

### Generation of eMPCs is impaired in mice that are deficient in GM-CSF

**Figure 5:**
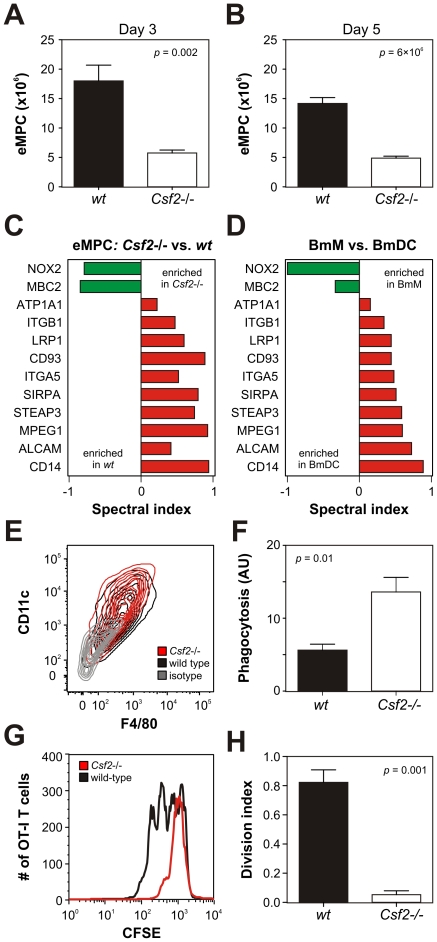
Analysis of eMPCs harvested from wild-type and GM-CSF-deficient (*Csf2−/−*) mice. eMPCs isolated from wild-type (*wt*) and *Csf2*−/− mice were interrogated for cell number, function, and protein expression. ***Panel A–B:*** Accumulation of eMPCs 3 days (*Panel A*) and 5 days (*Panel B*) following intraperitoneal injection with thioglycolate. Results (N = 6) are means and SEMs. ***Panel C:*** Plasma membrane proteomic analysis of eMPCs isolated from *Csf2*−/− and wild-type mice. Differentially-expressed proteins were identified using the *t-*test and *G-*test (*p*<0.05 and *G-*statistic >1.5) and quantified using the spectral index. ***Panel D:*** Proteins differentially expressed by eMPCs isolated from *Csf2*−/− mice (see *Panel C*) were measured in BmMs and BmDCs and quantified using the spectral index. ***Panel E:*** Cell surface CD11c and F4/80 expression on eMPCs was assessed by flow cytometry. Results are presented as contour plots with 10% probability increments. ***Panel F:*** Phagocytosis of fluorescein-labeled *E. coli* by eMPCs. Results (arbitrary units, AU; N = 4) are means and SEMs. ***Panel G–H:*** Antigen cross-presentation by eMPCs. Ovalbumin (0.2 mg/mL)-treated eMPCs were incubated with CFSE-labeled spleen cells isolated from OT-I transgenic mice. Levels of CFSE were assessed in OT-I T cells selected by flow cytometry and expression levels of CD8 and Vb5 (*Panel G*). The division index was calculated using FlowJo software. Results (N = 4) are means and SEMs (*Panel H*). Where applicable, *p*-values were derived using a two-tailed Student's *t-*test. Results obtained for eMPC quantification, flow cytometry, phagocytosis and antigen cross-presentation are representative of 3 independent analyses.

The overlap between the surface proteomes of cultured BmDCs generated with GM-CSF and eMPCs raises the possibility that GM-CSF contributes to the generation of eMPCs *in vivo*. To test this hypothesis, we injected thioglycolate into peritonea of wild-type and *Csf2*−/− (GM-CSF-deficient) mice and harvested cells 3 and 5 days later. We observed 80% fewer cells in the *Csf2*−/− mice than in the wild-type mice at both time points (**Fig. 5A–B**; day 3, *p* = 0.002; day 5, *p* = 6×10^−6^), suggesting that GM-CSF helps generate eMPCs in this model of sterile inflammation.

We next compared the plasma membrane proteomes of eMPCs from *Csf2*−/− and wild-type mice. Dual statistical criteria (*G-*test<−1.5 or >1.5, *t-*test: *p*<0.05) identified 16 proteins that were differentially expressed between wild-type and *Csf2*−/− eMPCs (**Table S5**). Interestingly, the expression levels of 75% (12 of 16) of these proteins in *Csf*2−/− eMPCs were consistent with a switch toward a more macrophage-like (i.e., M-CSF-driven) phenotype (**Fig. 5C–D**). In contrast, eMPCs isolated from *Csf2*−/− and wild-type mice expressed similar levels of F4/80 and CD11c, which are commonly used to differentiate macrophages from DCs (**Fig. 5E** **, Fig. S1**).

We assessed the functional properties of eMPCs harvested from wild-type and *Csf2*−/− mice. eMPCs isolated from *Csf2*−/− mice were significantly more efficient at phagocytosing fluorescein-labeled *E. coli* (**Fig. 5F**; 2.5-fold increase; *p* = 0.01, Student's *t-*test). In contrast, they were less able to cross-present antigen (**Fig. 5G–H**; 15-fold reduction; *p* = 0.001, Student's *t-*test). Taken together, these findings suggest that GM-CSF regulates both the number and functional attributes of eMPCs in this model of sterile inflammation.

## Discussion

**Figure 6:**
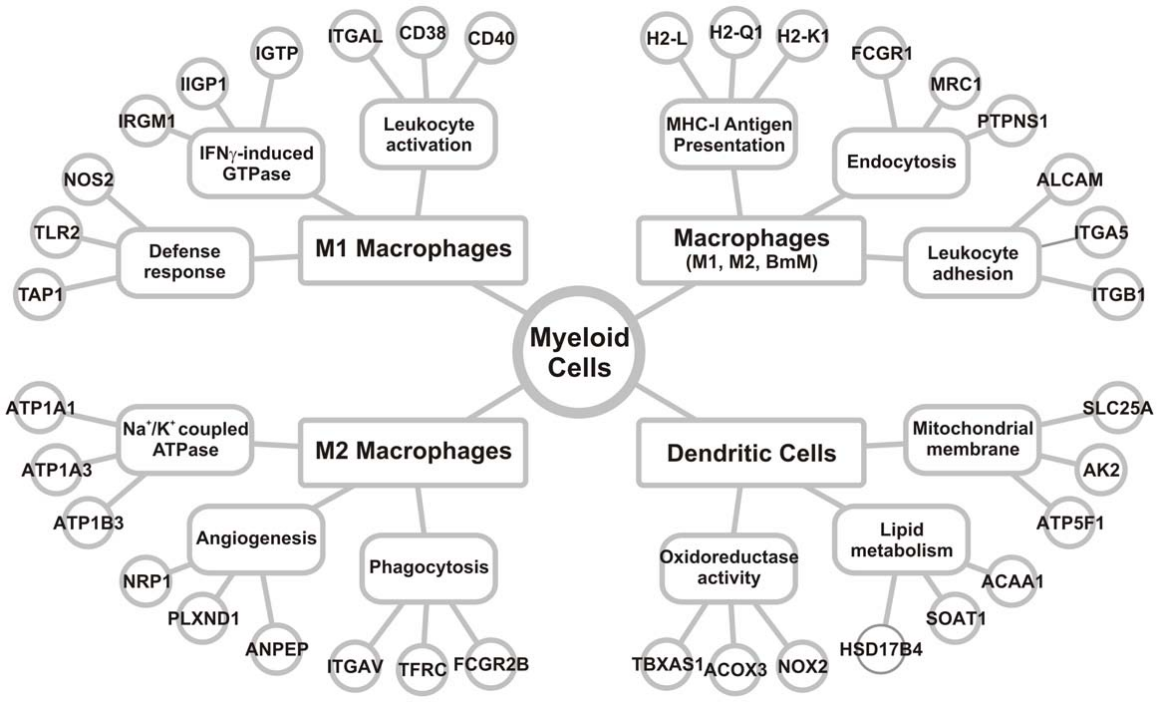
Plasma membrane protein signatures of myeloid cells identify unique cell functions. Gene ontology analysis of plasma membrane proteins enriched in M1 macrophages, M2 macrophages, all macrophage types (BmM, M1, and M2), and BmDCs identifies functional categories of proteins enriched in each cell type (*p*<0.05 with Benjamini-Hochberg correction). The top three functional annotations are presented for each cell type along with three representative proteins.

We used mass spectrometry to provide a global view of the proteomes of macrophages and DCs generated *in vitro* with M-CSF or GM-CSF. Using stringent dual statistical criteria, we identified 106 proteins that were enriched in the membranes of the different cell types generated *in vitro*. We also identified core sets of proteins that distinguished all the macrophage types and DCs from each other (**Fig. 6**).

Our data suggest that proteomics can distinguish macrophage classes from each other and from DCs. They are also consistent with the view that M-CSF and GM-CSF are major determinants of the polarization/differentiation of myeloid cells *in vivo* [24]. Moreover, they provide direct *in vivo* evidence that GM-CSF is a major phenotypic determinant of peritoneal myeloid cells of mice challenged with thioglycolate, a classic model of sterile inflammation.

Certain of the proteins we identified are widely used as markers of cell phenotype *in vivo*. For example F4/80 is generally regarded as a marker for macrophages [7], and our proteomic analyses detected much higher levels of F4/80 in the plasma membrane of macrophages than in that of DCs. We also found that some widely used markers are not specific. One striking example is CD11c – often used as a DC marker. Mass spectrometry and immunofluorescence detected high levels of CD11c in the plasma membrane of M2 macrophages. Indeed, recent findings demonstrate that CD11c fails to discriminate between macrophage and DC populations *in vivo* [25]. However, most of the proteins that were enriched in the membranes of the various cell types have not been described previously.

Because plasma membrane proteins are central to the critical functions of macrophages and DCs, our findings suggest that unique plasma membrane protein expression patterns not only interrogate the phenotypes of myeloid cells but also form an important basis for the cells' distinct functional properties. Thus, over 50% of the plasma membrane proteins we identified were selectively expressed by one of the four myeloid cell types, supporting the conclusion that polarized macrophages and DCs fulfill distinct biological roles (**Fig. 6**).

*In vivo*, the phenotypes of macrophages and DCs are regulated by complex, dynamic tissue environments, making it unlikely that a limited number of markers can define myeloid cell heterogeneity. Indeed, it is generally accepted that the utility of widely used myeloid markers such as F4/80, CD11b, and CD11c is highly tissue-dependent [9], [26], [27]. Proteomic signatures, on the other hand, should be more effective tools for defining cell phenotype because patterns of protein expression are more resistant than single measurements to the highly complex and variable environments macrophages and DCs encounter *in vivo*.

The power of using protein expression patterns to establish myeloid cell phenotypes is highlighted by the remarkable similarities we observed between the plasma membrane proteome of DCs generated with GM-CSF and that of eMPCs isolated from the inflamed peritoneum of mice. Similar findings were obtained when BmDCs and eMPCs were clustered according to gene expression patterns obtained from a meta-analysis of *in vitro* and *in vivo* generated myeloid cells [28]. We also found that markedly fewer ePCs accumulate in GM-CSF-deficient (*Csf2*−/−) mice. Functional characterization showed that cells isolated from *Csf2*−/− mice were less able than those from wild-type mice to cross-present antigen (a function classically ascribed to DCs) and more able to phagocytose bacteria (a function classically ascribed to macrophages).

These observations strongly suggest that correlates of the myeloid DCs generated with GM-CSF *in vitro* also exist *in vivo*. Moreover, they are consistent with previous studies showing that *i*) thioglycolate induces peritoneal myeloid cells to express and secrete GM-CSF [29], *ii*) thioglycolate-elicited peritoneal myeloid cells emigrate to draining lymph nodes as inflammation resolves—a classic property of DCs [9], [30], and *iii*) thioglycolate-elicited peritoneal cells can present antigens and stimulate T cell proliferation [31].

Our proteomic analyses, together with functional analyses of macrophages and DCs by many investigators [1]–[3], [7], depict a scenario in which M-CSF and GM-CSF support highly polarized protein expression patterns that influence diverse biological endpoints ranging from antigen presentation to cell motility, phagocytosis, generation of reactive oxygen species, fatty acid oxidation, and inflammation. Moreover, previous studies have validated many of the functional differences predicted by our proteomic data. For example, NADPH oxidase, a protein over-expressed by BmDCs, has been assigned a key role in antigen presentation by DCs *in vivo* [32]. Importantly, changes in the tissue milieu would elicit corresponding changes in resident and recruited myeloid cells. Consequently, cell types with customized patterns of protein expression (and hence function) would be generated, and such cells would be well-suited to meet the dynamic demands of the local environment.

Collectively, our observations provide a rich proteomic framework that should help investigators identify specific populations of macrophages and DCs in tissue so they can correlate functions with the correct cellular phenotypes. By using patterns of protein expression specific to each type of myeloid cell, it will now be possible to more confidently extrapolate data from *in vitro* experiments to more complex *in vivo* situations. As myeloid-derived cells are implicated in autoimmune diseases, cancer, infection, and many other conditions, our membrane proteome signatures should help investigators identify the specific populations that are central to the pathogenesis of those disorders.

## Materials and Methods

### Ethics Statement

All animal studies were approved by the Institutional Animal Care and Use Committee (IACUC) at the University of Washington (protocol 3437-01).

### Differentiation of myeloid cells

Macrophages and DCs were derived from bone marrow of C57BL/6 mice [19], [33]. For proteomic analyses, bone marrow-derived cells from the tibias and fibulas of 5 mice were pooled and plated on day 0 in T75 flasks (15×10^6^ cells per flask; Corning). For mRNA quantification or confocal microscopy, 2×10^6^ or 2.5×10^4^ bone marrow-derived cells were plated into 6-well plates (Corning) or 8-well chamber slides (Nunc), respectively. BmDCs were cultured at 37°C in 5% CO_2_ in RPMI medium containing 10% FBS and 10 ng/mL of GM-CSF (R&D Systems). Macrophages were cultured in low D-glucose (1 g/L) Dulbecco's minimum essential medium (DMEM) supplemented with 10% FBS and 30% L cell-conditioned medium [34]. Medium was replaced on days 2 and 4 (with retention of floating and attached cells) and on day 6, when floating cells were discarded. BmMs and BmDCs were harvested on day 7. To induce M1 or M2 phenotypes, BmMs were stimulated for 24 h with IFN-γ (12 ng/mL; R&D Systems) and LPS (5 ng/mL; Sigma) or for 48 h with IL4 (10 ng/mL; R&D Systems).

### Collection of thioglycolate-elicited peritoneal cells

C57BL/6J-*Ldlr^tm1Her^*, *Csf2*−/−, and wild-type mice on the C57Bl/6J genetic background were injected with thioglycolate (Sigma), and cells were harvested from the peritoneal cavity 5 days after injection. The cells were washed with phosphate-buffered saline (PBS), plated, and allowed to adhere at 37°C for 2 h in serum-free DMEM. At the end of that period, cells were washed 3 times with PBS to remove non-adherent cells. eMPC numbers in the peritonea of wild-type (N = 6) and *Csf2*−/− (N = 6) mice 3 or 5 days following thioglycolate administration were determined by counting CD11b^HI^ expressing cells by flow cytometry.

### Isolation of plasma membrane-associated proteins

Cell-surface proteins were isolated, using a membrane-impermeable, cleavable biotinylation reagent (N-hydroxysulfosuccinimide-SS-biotin; Pierce) to label primary amines of proteins [35]. Briefly, cells were biotinylated at 4°C for 1 h, harvested, and lysed. Cell lysates were passed over neutravidin agarose resin, and the retained proteins were eluted with 100 mM DTT. In parallel, non-biotinylated cells were subjected to the same procedure to identify proteins that bound nonspecifically to the resin. Eluted proteins were alkylated with 125 mM iodoacetamide, and digested overnight at 37°C with sequencing-grade trypsin (1∶50, w/w, trypsin/protein; Promega). Tryptic digests were mixed with acetic acid (1∶1, v/v), and extracted on a C18 column (HLB, 1 mL; Waters Corp.) according to the manufacturer's protocol. Fractions containing peptides were dried under vacuum and resuspended in 0.3% acetic acid/5% acetonitrile (1 mg protein/mL).

### Liquid chromatography-electrospray ionization-tandem MS (LC-ESI-MS/MS)

Tryptic digests (2 µg protein) were injected into a trap column (Paradigm Platinum Peptide Nanotrap, 0.15×50 mm; Michrom Bioresources, Inc.) and desalted for 5 min with 5% acetonitrile, 0.1% formic acid (50 µL/min). Peptides were eluted onto an analytical reverse-phase column (0.150×150 mm, 5 µm beads; Magic C18AQ, Michrom Bioresources, Inc.) and separated at a flow rate of 1 µL/min over 180 min, using a linear gradient of 5% to 35% buffer B (90% acetonitrile, 0.1% formic acid) in buffer A (5% acetonitrile, 0.1% formic acid). Mass spectra were acquired in the positive ion mode, using electrospray ionization and a linear ion trap mass spectrometer (LTQ, Thermo Electron Corp.) with data-dependent acquisition. MS/MS scans were obtained on the 8 most abundant peaks in each survey MS scan.

### Peptide and protein identification

MS/MS spectra were searched against the mouse International Protein Index (IPI) database (version, 2007/10/30) [36], using the SEQUEST search engine with the following search parameters: unrestricted enzyme specificity, 2.8 amu precursor ion mass tolerance, 1.0 amu fragment ion mass tolerance, fixed Cys alkylation, and variable Met oxidation. SEQUEST results were further validated with PeptideProphet [14] and ProteinProphet [15], using an adjusted probability of ≥0.90 for peptides and ≥0.96 for proteins. Proteins considered for analysis had to be identified in at least 5 (of 6) biological replicates of at least one cellular phenotype. When MS/MS spectra could not differentiate between protein isoforms, all were included in the analysis.

### Protein quantification and statistical analysis

Proteins detected by LC-ESI-MS/MS were quantified by spectral counting (the total number of MS/MS spectra detected for a protein [16]). Differences in relative protein abundance were assessed with the *t-*test and *G-*test [17]. Permutation analysis was used to empirically estimate the FDR [37]. Significance cutoff values for the *G-*statistic and *t-*test were determined using PepC [18], a software package that maximizes the number of differentially expressed proteins identified for a given FDR.

### Hierarchical clustering

Spectral counts for each protein in each sample were normalized to mean expression across all cell types, using (SC_X,N_−SC_X,AVG_)/(SC_X,N_+SC_X,AVG_), where SC_X,N_ represents the spectral counts for a given protein (X) in a given analysis (N) and SC_X,AVG_ is the average spectral count for that protein across all analyses. This equation normalizes relative protein expression to a value between −1 and +1 [38]. Hierarchical clustering of samples and proteins was performed using MultiExperiment Viewer [39] with Pearson correlation as the distance metric and average linkage clustering as the linkage method.

### Functional annotation

Functional enrichments in Gene Ontology annotations in the plasma membrane-associated proteome (relative to the entire mouse genome) were identified using the Bingo 2.0 plugin in Cytoscape (V2.5.2) [40]. Statistical significance was assessed using the hypergeometric test with Benjamini-Hochberg correction [37].

### qRT-PCR

To quantify mRNA expression, total RNA was isolated from cells, using TRIzol Reagent (Invitrogen) and the RNeasy Mini Kit (Qiagen). Total RNA (1 µg) was reverse transcribed, using the iScript Select cDNA Synthesis Kit (Bio-Rad). The sequences for sense strand and antisense strand PCR primers are provided in Supplementary Material (**Table S6**). PCR amplification of cDNA samples was performed using SensiMix SYBR (Bioline) on a 7900HT Fast Real-Time PCR system (Applied Biosystems). Relative quantification of PCR products was based on value differences between the target and 18S control, using the 2−ΔΔCT method [41].

### Confocal microscopy

Macrophages, BmDCs, and eMPCs grown in 8-well chamber slides (Nunc) were formalin-fixed (10 min at room temperature), washed with PBS, and blocked with 1% bovine serum albumin (BSA) overnight at 4°C. Cells were probed with primary antibodies (0.5–1 µg/mL) in PBS containing 0.1% BSA for 2 h at room temperature. Antibodies against murine CD11a (Abcam), CD11b (Abcam), CD11c (Abcam), CD40 (Abcam), MBC2 (Abcam), F4/80 (Abcam), ITGA6 (Abcam), ITGB5 (Abcam), MAC2 (Cedarlane), FER1L (Abcam), STAB1 (Santa Cruz), and TFRC (Abcam) were used in this study. Slides were incubated with appropriate TRITC-labeled secondary antibodies (1∶500; Molecular Probes), mounted in medium containing DAPI (Vector), and visualized with a Nikon A1 confocal microscope. For each antibody, all analyses of cells were performed on the same day with identical exposure settings on the microscope. We were careful to avoid signal saturation for the cell type with the highest expression (based on proteomic quantification) of each protein. Appropriate isotype controls tested specificity.

### Flow cytometry

In some cases, cells were detached from culture plates with 8 mg/mL lidocaine and 5 mM EDTA for 10 min at 37°C. Cells were incubated with specific antibodies for 30 min at 37°C with the following fluorophore-labeled primary antibodies (eBioscience): MHC class II-APC (I-A/I-E, 25 ng/10^6^ cells), F4/80-FITC (250 ng/10^6^ cells), CD11b-PB (75 ng/10^6^ cells), and CD11c-PE (150 ng/10^6^ cells). Cells were sorted with a FACS Canto (BD Biosciences), and the data were analyzed with FlowJo software (V. 8.8.6, Tree Star, Inc.). Appropriate isotype controls tested specificity. FACS analyses represent all live cells as assessed by propidium iodide or calcein blue staining (eBioscience).

### Antigen cross-presentation assays

Thioglycolate-elicited peritoneal cells (0.5×10^6^/well; 24-well plate, NUNC) isolated from wild-type or *Csf2−/−* mice were incubated overnight at 37°C in serum-free DMEM containing 10-fold serial dilutions of ovalbumin (2 mg/ml, 0.2, 0.02, and 0.002 mg/mL). At that time, macrophages were washed and incubated with CFSE-labeled spleen cells (5×10^6^/well) isolated from OT-I transgenic mice. Cells were co-cultured in RPMI supplemented with 10% FCS, 50 µM β-ME for 3 days. OT-I T cells were gated based on CD8 and Vb5 expression, and CFSE dilution was analyzed by flow cytometry. The division index was calculated using FlowJo software (V. 8.8.6, Tree Star, Inc.).

### Phagocytosis assays

Phagocytosis of fluorescein-labeled *E. coli* bioparticles was assessed using the Vibrant™ Phagocytosis Assay Kit (Invitrogen), as previously described [42].

## Supporting Information

Figure S1 **Flow cytometric analysis of myeloid cells.** ***Panel A:*** Bone marrow-derived macrophages (BmM) and dendritic cells (BmDCs) were obtained by culturing bone marrow cells with M-CSF and GM-CSF respectively. Flow cytometric analysis of CD11c, F4/80, and MHC-II expression in BmDCs and BmMs. Results are directly comparable to **Figure 2B–C** in the main manuscript. ***Panel B:*** F4/80, CD11b, and CD11c expression in thioglycolate-elicited myeloid peritoneal cells (eMPC) isolated from C57BL/6 mice. Results are directly comparable to **Figure 4D–E** in the main manuscript. ***Panel C:*** Cd11c and F4/80 expression in ePMCs isolated from wild-type or *Csf2*−/− (GM-CSF-deficient) mice. Results are directly comparable to **Figure 5E** in the main manuscript. Where applicable, results are presented as contour plots with 10% probability increments.
(TIF)

Figure S2 **The plasma membrane proteome classifies myeloid cells.** ***Panels A–B:*** Hierarchical cluster analysis. Spectral counts for each protein (192 total) in each cell type were normalized to the mean expression level across all four cell types and analyzed by hierarchical clustering with Pearson correlation as the distance metric and average linkage clustering as the linkage method. Red = overexpression; green = underexpression. ***Panel B:*** Demonstration of clustering of cell types more clearly.
(TIF)

Figure S3 **Immunocytochemical detection of plasma membrane protein markers.** Expression levels of newly identified markers of M1 cells, M2 cells, BmMs and BmDCs was assessed by mass spectrometry (***Panel A***) and immunocytochemistry (***Panel B***). For MS/MS, proteins were quantified by spectral counting and expressed relative to the cell type with the highest expression level for each protein. Results are means and SDs. Cells were stained with antibodies specific to each protein (red channel) and counterstained with DAPI to visualize nuclei (blue-channel) and examined by confocal microscopy. Immunostaining and microscopy were performed on the same day with identical microscope settings. Results are representative of 3 independent analyses.
(TIF)

Table S1 **Plasma membrane proteins detected by LC-ESI-MS/MS in myeloid cells.** Bone marrow precursor cells were differentiated into bone marrow-derived macrophages (BmM), classically-activated macrophages (M1), alternatively-activated macrophages (M2), and bone marrow-derived dendritic cells (BmDC). Plasma membrane proteins for each cell type (N = 6) were isolated, analyzed by mass spectrometry, and quantified by spectral counting, the total number of peptides identified by LC-ESI-MS/MS analysis.
(XLSX)

Table S2 **Identification of plasma membrane proteins that distinguish amongst polarized macrophages.** Protein markers specific for each macrophage population tested (or signatures) were identified based on both the *t-*test (*p*-value) and *G-*test (*G-*statistic). Protein markers for a given cell type were defined as those that were consistently up-regulated (*G*>1.5 and *p*<0.05; red) or down-regulated (*G*<−1.5 and *p*<0.05; green) relative to all macrophage cell types tested. Statistical significance cutoffs for the *G-*test and *t-*test were established by random permutation analysis to ensure that the false-discovery rate <5%.
(XLSX)

Table S3 **Plasma membrane proteomics of bone marrow-derived dendritic cells (BmDC).** Overlaps between plasma membrane protein expression in BmDCs and the macrophage signatures identified (see **Fig. 1** and **Table S2**), as well as the BmDC signature were identified based on the *t-*test and *G-*test. Protein markers of BmDCs (BmDC signature) were defined as those that were consistently up-regulated (*G*>1.5 and *p*<0.05; red) or down-regulated (*G*<−1.5 and *p*<0.05; green) relative to all macrophage populations tested.
(XLSX)

Table S4 **Plasma membrane proteomics of thioglycolate-elicited myeloid peritoneal cells (eMPCs).** Overlaps between plasma membrane protein expression in eMPCs and BmMs, M1, M2, and BmDCs (highlighted in blue) were identified based on the *t-*test and *G-*test. For example, overlaps between eMPCs and the BmDC signature were defined as those proteins for which expression in eMPCs (like BmDCs) is consistently up-regulated (*G*>1.5 and *p*<0.05; red) or down-regulated (*G*<−1.5 and *p*<0.05; green) relative to all macrophage populations tested.
(XLSX)

Table S5 **Plasma membrane proteins differentially expressed in** ***Csf2*** **−/− mice.** eMPCs isolated from wild-type (*wt*) and *Csf2*−/− mice were analyzed by mass spectrometry. Differentially expressed proteins were identified by the *t-*test (*p*<0.05) and *G-*test (*G*>1.5 or <−1.5). Red = up-regulated and green = down-regulated in eMPCs isolated from *Csf2*−/− relative to *wt* mice.
(XLSX)

Table S6 **PCR primers used in this study.**
(XLSX)
