# Supplementary figures and images for: Unique Proteomic Signatures Distinguish Macrophages and Dendritic Cells
Source: PLoS One. 2012 Mar 12;7(3):e33297. doi: 10.1371/journal.pone.0033297 (PMC3299764; doi:10.1371/journal.pone.0033297)

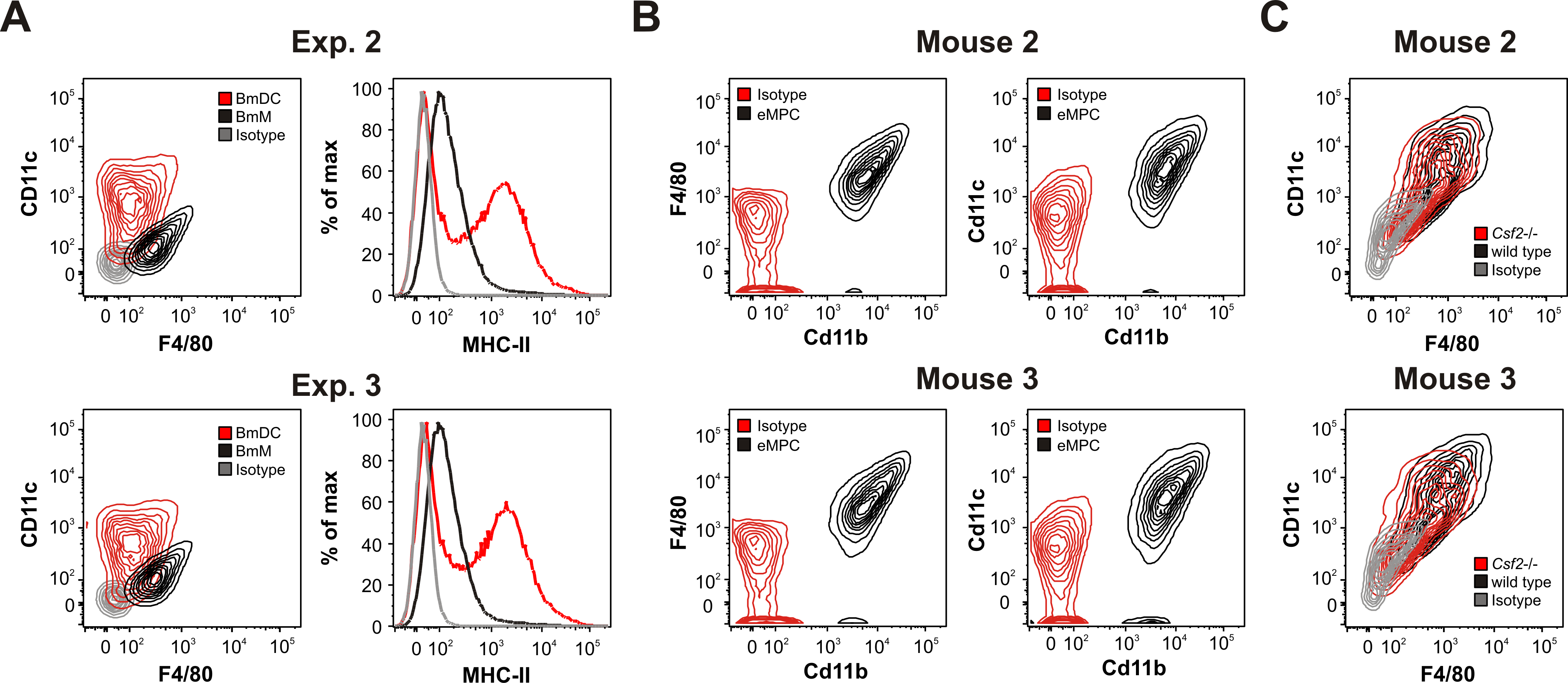

Supplement: Figure S1 — Flow cytometric analysis of myeloid cells. Panel A: Bone marrow-derived macrophages (BmM) and dendritic cells (BmDCs) were obtained by culturing bone marrow cells with M-CSF and GM-CSF respectively. Flow cytometric analysis of CD11c, F4/80, and MHC-II expression in BmDCs and BmMs. Results are directly comparable to Figure 2B–C in the main manuscript. Panel B: F4/80, CD11b, and CD11c expression in thioglycolate-elicited myeloid peritoneal cells (eMPC) isolated from C57BL/6 mice. Results are directly comparable to Figure 4D–E in the main manuscript. Panel C: Cd11c and F4/80 expression in ePMCs isolated from wild-type or Csf2−/− (GM-CSF-deficient) mice. Results are directly comparable to Figure 5E in the main manuscript. Where applicable, results are presented as contour plots with 10% probability increments. (TIF) [file pone.0033297.s001.tif]

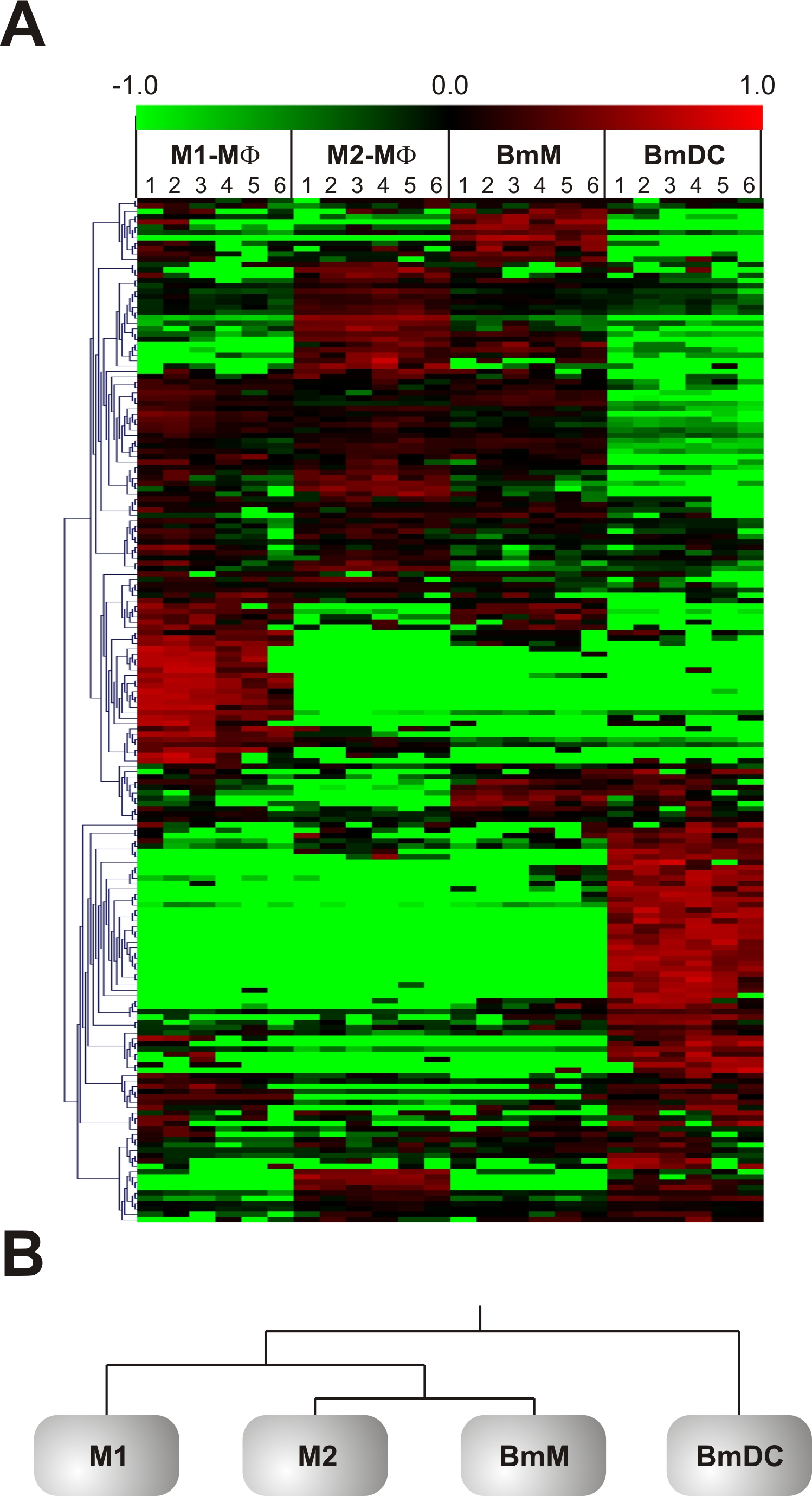

Supplement: Figure S2 — The plasma membrane proteome classifies myeloid cells. Panels A–B: Hierarchical cluster analysis. Spectral counts for each protein (192 total) in each cell type were normalized to the mean expression level across all four cell types and analyzed by hierarchical clustering with Pearson correlation as the distance metric and average linkage clustering as the linkage method. Red = overexpression; green = underexpression. Panel B: Demonstration of clustering of cell types more clearly. (TIF) [file pone.0033297.s002.tif]

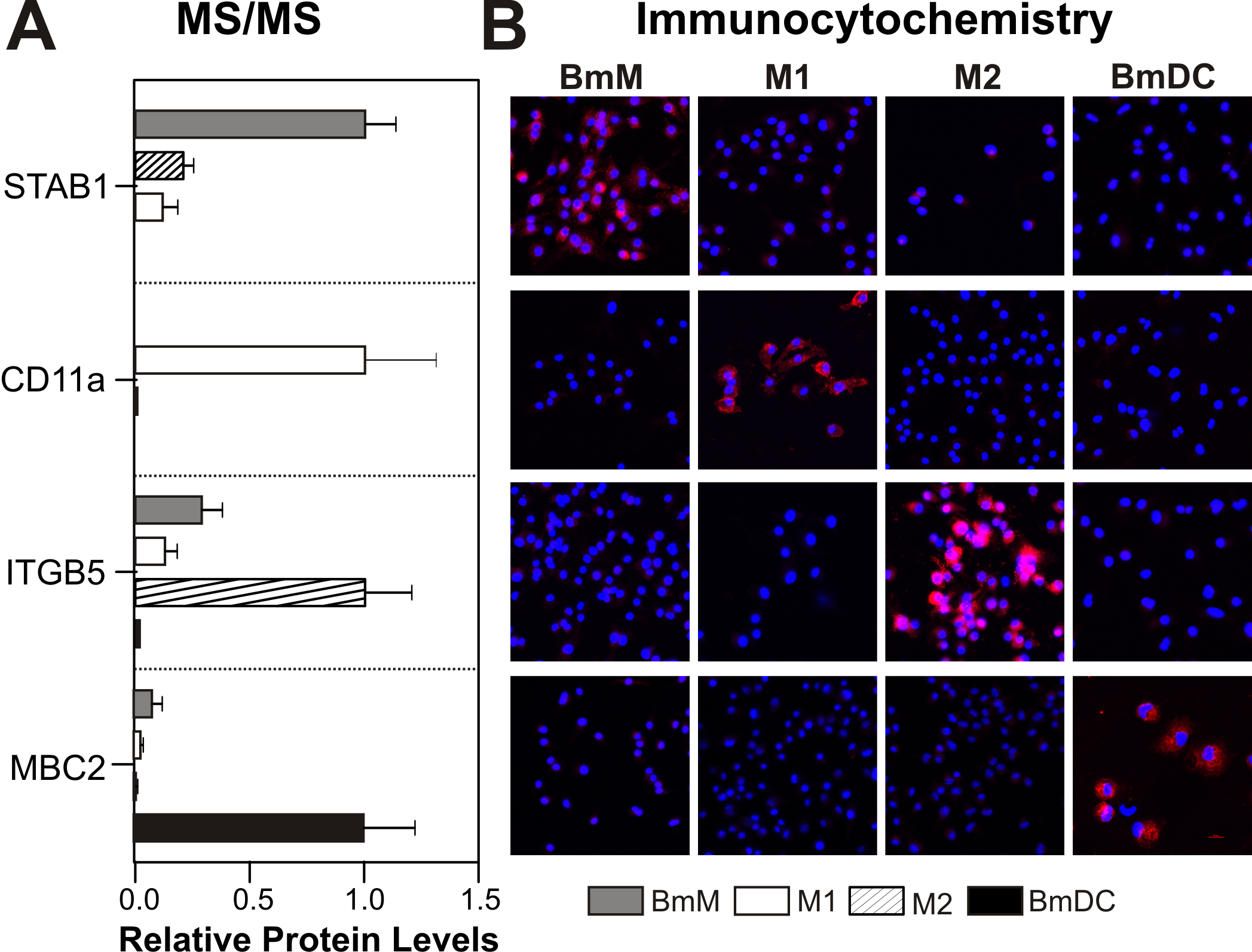

Supplement: Figure S3 — Immunocytochemical detection of plasma membrane protein markers. Expression levels of newly identified markers of M1 cells, M2 cells, BmMs and BmDCs was assessed by mass spectrometry (Panel A) and immunocytochemistry (Panel B). For MS/MS, proteins were quantified by spectral counting and expressed relative to the cell type with the highest expression level for each protein. Results are means and SDs. Cells were stained with antibodies specific to each protein (red channel) and counterstained with DAPI to visualize nuclei (blue-channel) and examined by confocal microscopy. Immunostaining and microscopy were performed on the same day with identical microscope settings. Results are representative of 3 independent analyses. (TIF) [file pone.0033297.s003.tif]
